# When Support Backfires: Narcissistic Self-Regulatory Strategies, Ego Threat, and Workplace Aggression

**DOI:** 10.3390/bs16040552

**Published:** 2026-04-07

**Authors:** Ryoichi Semba

**Affiliations:** Faculty of Business Administration, Kyoto Tachibana University, 34 Yamada-cho Oyake Yamashina-ku, Kyoto 607-8175, Japan; semba@tachibana-u.ac.jp; Tel.: +81-75-571-1111

**Keywords:** ego threat, supervisor support, organizational support, power harassment, workplace aggression, narcissistic type, narcissism, self-regulatory strategy

## Abstract

Although ego threat is known to influence workers’ aggressive behavior, little is understood about how support and narcissism shape this relationship. Accordingly, the present study conceptualized narcissistic traits as distinct self-regulatory strategies for maintaining self-worth and examined whether the meaning of support under ego threat varies depending on these traits. An online survey was conducted with 1621 Japanese workers, and the participants were classified into three types—Self-Assertion, Need for Attention and Praise, and Sense of Superiority and Competence—based on the highest scores on the three factors of the Narcissistic Personality Inventory Short version. Hierarchical multiple regression analyses were then conducted separately for each type. The results showed that the behavioral consequences of ego threat varied substantially across narcissistic types and that support did not uniformly suppress power harassment. For the Self-Assertion type, perceived organizational support was positively associated with Invasion of Privacy. For the Need for Attention and Praise type, men and managers tended to choose Excessive Demands. For the Sense of Superiority and Competence type, supervisor support reduced harassment; however, under strong ego-threatening conditions, such support paradoxically amplified harassment. These findings suggest that support functions as a socially meaningful cue whose interpretation depends on narcissistic self-regulatory strategies.

## 1. Introduction

In contemporary workplaces, interpersonal problems have become increasingly serious, contributing to heightened psychological stress among workers and declines in organizational functioning. According to the Japanese MHLW (2023), 82.2% of employees report experiencing strong anxiety, stress, or worries related to their work or professional life, with one major source being “interpersonal relationships (including sexual and power harassment)” (26.2%). Furthermore, a report by the Japanese MHLW (2025) indicates that among the 267,755 individual labor dispute consultations handled nationwide in the fiscal year 2024, the most frequent category was “bullying and harassment” (54,987 cases), suggesting a continuing increase in power harassment.

Power harassment is a concept developed in Japan that is defined as “behavior in the workplace based on a superior relationship that goes beyond what is necessary and appropriate for work and harms the working environment of employees” (MHLW, 2012). Although this concept is used within a uniquely Japanese institutional context, its substantive content substantially overlaps with counterproductive behavior and organizational dysfunctional behavior examined in Western organizational research (Semba & Haraguchi, 2014). In particular, concepts such as abusive supervision (Tepper, 2000), workplace bullying (Einarsen et al., 2010), and mobbing (Leymann, 1996) share common features, including the abuse of hierarchical power, repeated negative acts, and detrimental effects on subordinates’ well-being and job performance.

Furthermore, a recent meta-analysis (Zhong et al., 2025) indicates that self-evaluation and the quality of interpersonal relationships play important roles in the processes through which workplace aggression affects employee outcomes, suggesting that these psychological mechanisms may have a certain degree of universality across cultures. Therefore, by positioning power harassment in Japan within the context of international research on workplace aggression, it is possible to clarify its broader theoretical significance while taking into account its cultural specificity.

In a rapidly aging society with a shrinking workforce such as Japan, it is increasingly important to create environments in which individuals can fully utilize their abilities. Accordingly, establishing support systems that prevent workers’ self-evaluations from being threatened—or, if threatened, from escalating into aggressive behavior—is critical. To develop effective strategies for reducing power harassment, it is first necessary to clarify its antecedents and construct a theoretical model that explains its occurrence. However, to date, few studies have integratively examined both the determinants of power harassment and their underlying theoretical framework. In contrast, substantial findings have accumulated regarding organizational dysfunctional behavior, a closely related concept.

Among the determinants of organizational dysfunctional behavior, narcissism has been identified as one of the most powerful explanatory variables (Tanaka, 2008). A prominent theoretical account of the relationship between narcissism and aggression is the threatened egotism model proposed by Baumeister et al. (1996). According to this model, individuals with high but unstable self-esteem experience ego threat when they receive negative evaluations from others because such evaluations conflict with their self-concept. To prevent a decline in self-esteem, they adopt coping strategies aimed at compensating for or maintaining their self-worth (Tabata & Ikegami, 2011), which may result in behaviors such as social withdrawal or aggression and violence.

Oshio (1998) further demonstrated that the three subscales of the Narcissistic Personality Inventory (hereafter NPI; Raskin & Hall, 1979) exert different effects on dimensions of self-esteem, suggesting that these narcissistic factors serve distinct functions in maintaining positive self-evaluation. Building on this perspective, Semba (2016a, 2018) used the three NPI (Narcissistic Personality Inventory) subscales to examine how narcissistic functions are involved in coping behaviors and behavioral choices in organizations. Semba (2016b) incorporated organizational support factors and narcissistic types into the threatened egotism model and analyzed the process through which workers move from ego threat to organizational dysfunctional behavior. The results indicated that supervisor support can reduce such behavior and that the perception and seeking of support differ by narcissistic type. Semba (2018) further demonstrated that both the type of organizational support and narcissistic type influence behavioral choices following ego threat.

Taken together, these findings suggest that power harassment, as a concept adjacent to organizational dysfunctional behavior, can also be understood as a defensive reaction to perceived ego threat. In particular, examining how supervisory and organizational support shapes the process from ego threat to aggressive behavior (i.e., power harassment) across narcissistic types is both theoretically and practically important.

More specifically, narcissism is a psychological function aimed at maintaining positive self-evaluation and is defined as the function “to maintain the cohesiveness, stability, and positive affective colouring of the self-representation” (Stolorow, 1975). It consists of three factors: Self-Assertion, Need for Attention and Praise, and Sense of Superiority and Competence (Oshio, 2004, 2006). Behavior is thought to be characterized by the relative strength of these factors; for example, individuals high in Self-Assertion emphasize active and independent interpersonal relations and may not require harmonious relationships (Oshio, 2006). Those high in Need for Attention and Praise tend to adopt passive and dependent interpersonal styles and may feel anger but refrain from expressing it (S. Abe & Takagi, 2006a, 2006b). Individuals high in Sense of Superiority and Competence are more likely to legitimize one-sided expressions directed at others, such as “doing things others dislike” or “making sarcastic remarks” (S. Abe & Takagi, 2006b).

A series of studies by Semba (2017, 2018, 2024, 2025) have shown that the relative strength of these factors influences subsequent behavior; for instance, Tanaka (2008) found that Self-Assertion positively predicted verbal violence and verbal harassment. Hibino et al. (2005) showed that Self-Assertion promoted aggressive behavior or displacement of anger onto objects through forced cognitive closure. Need for Attention and Praise was found to promote aggression via anger (Hibino et al., 2005), and Sense of Superiority and Competence showed age-dependent relations with verbal aggression (Sagara & Sagara, 2006).

Narcissism is also closely related to self-esteem. Because maintaining positive self-evaluation requires a sense of acceptance from others (Leary & Baumeister, 2000), social support plays a central role. Social support generates the perception of being accepted and valued (Cobb, 1976; Genjida, 2012), which helps stabilize self-worth and reduce defensive reactions to ego threat.

However, support does not always function beneficially. The perception of support under ego threat differs by psychological characteristics (Kato et al., 2013). Some individuals feel encouraged, whereas others experience support as further threatening. Court cases indicate that conflicts often arise from supervisory guidance and training (Japan Institute for Women’s Empowerment & Diversity Management, 2011). Moreover, receiving help can induce feelings of indebtedness and self-threat (Nadler & Fisher, 1986).

Given that support operates at both the interpersonal and organizational levels, the present study examines both supervisory and organizational support. Perceived organizational support refers to employees’ beliefs regarding the extent to which the organization values their contributions and cares about their well-being (Eisenberger et al., 1986). Such support conveys positive messages that can enhance self-evaluation (Elloy & Randolph, 1997).

Furthermore, in light of Japan’s interdependent and hierarchical cultural context, where harmony and role positioning are emphasized (Markus & Kitayama, 1991; Heine et al., 1999), support may be interpreted as evaluation rather than mere assistance. Accordingly, its effects are likely to interact with both narcissistic self-regulation and cultural norms.

Despite these theoretical and empirical advances, an important research gap remains. Although previous studies have examined narcissism as a predictor of aggressive behavior and dysfunctional behavior in organizations, few have integratively explored how ego threat, perceived support, and narcissistic self-regulatory strategies interact to influence the occurrence of power harassment. Furthermore, the dual role of support—namely, as a protective resource that inhibits aggressive behavior and as a situational cue that may intensify defensive reactions—has not been sufficiently examined empirically. This issue is particularly underexplored in hierarchical organizational contexts such as Japan. Addressing this gap is essential for understanding both the conditions under which support buffers aggressive behavior and those under which it may paradoxically promote dominance-oriented responses.

Based on this review of the literature, the present study aims to apply the analytic framework of organizational dysfunctional behavior research to examine how perceived ego threat influences power harassment and how perceived support from supervisors and organizations moderates this relationship among Japanese workers, with particular attention to differences across narcissistic types.

Based on the above theoretical framework, the present study proposed the following hypotheses. First, drawing on the ego threat model, which posits that aggressive or dominance-oriented behaviors arise as a means of defending a threatened self, ego threat is expected to be positively associated with power harassment behavior (Hypothesis 1). Second, because social support may function as a psychological resource that stabilizes self-evaluation, support from supervisors is expected to moderate the relationship between ego threat and power harassment (Hypothesis 2). Third, support from the organization may also influence how employees respond to ego threat; therefore, it is expected to moderate the relationship between ego threat and power harassment (Hypothesis 3). Finally, because narcissism reflects individual differences in self-regulatory strategies for maintaining self-evaluation, the effects of ego threat and support on power harassment are expected to differ across narcissistic types (Hypothesis 4).

## 2. Materials and Methods

A cross-sectional questionnaire survey was conducted among Japanese workers to examine the relationships among ego threat, perceived support, narcissistic types, and power harassment behavior. This methodological approach was chosen because the study aimed to assess workers’ subjective perceptions of psychological states such as ego threat and perceived support, as well as their self-reported behavioral tendencies within organizational contexts. These constructs are difficult to observe directly and are typically measured using standardized self-report scales in organizational and social psychology research.

### 2.1. Research Flow

**Figure 1:**
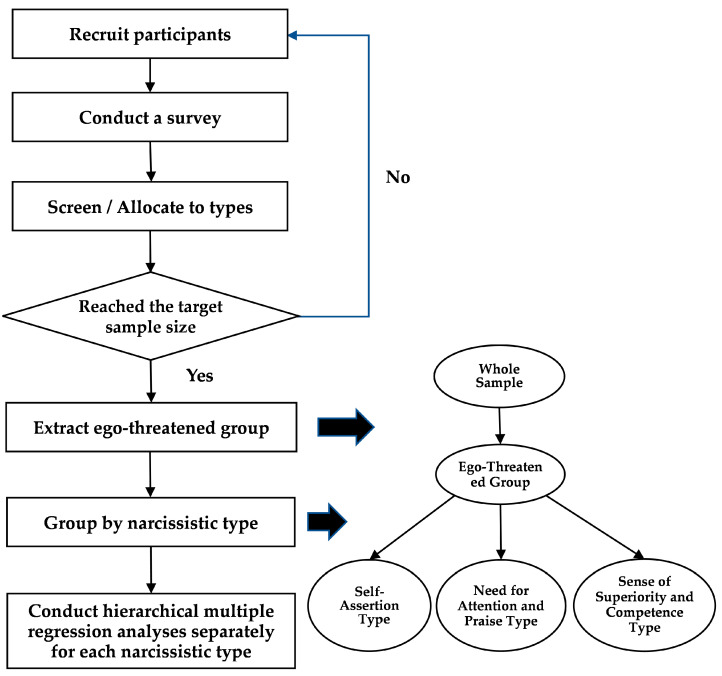
Research flow.

This survey was conducted in May 2024 through an online survey platform operated by Company A. Company A recruited respondents and administered the questionnaire survey. The collected data were screened to select cases that met the quality control criteria (i.e., passed attention checks), could be classified by narcissistic type (i.e., among the three subscales of the Narcissistic Personality Inventory Short version (Oshio, 1998) (hereafter NPI-S), only one had the highest score), and contained no missing values. Qualified cases were then distributed into each quota group. This process was repeated across all quotas—defined by gender, age, and narcissistic type—until the target sample size was achieved. The sample was then divided into high and low groups based on the median of the State Self-Esteem Scale; the high group was defined as the “ego-threatened group.” Finally, the ego-threatened group was classified into three groups based on narcissistic type and hierarchical multiple regression analyses were conducted separately for each group (see Figure 1).

Informed consent was obtained through Company A’s system by presenting participants with an explanatory statement describing the research purpose, voluntary nature of participation, right to withdraw, and procedures for the handling of personal information (see Appendix A). Participants provided consent by checking a box (responses could not be submitted without providing consent). All data were anonymized by Company A, and no personally identifiable information (e.g., IP addresses) was disclosed to the author.

To ensure data quality, additional screening procedures were implemented. Specifically, only respondents who were currently employed were included in the analysis. Furthermore, responses with substantial missing data or lacking consistency—such as those showing contradictions across related items—were excluded. As a result, only responses that met all of these criteria were used in the subsequent analyses.

The study was conducted in accordance with the Declaration of Helsinki, and approved by the Ethics Committee of Kyoto Tachibana University (Approval No. 24-11, dated 13 May 2024).

The dataset of the sample is available in the Hiroshima University Institutional Repository (accession number 2041434) at https://hiroshima.repo.nii.ac.jp/records/2041434 (accessed on 27 January 2026).

In the preparation of this manuscript, generative artificial intelligence (ChatGPT, version 4.1) was used solely for the translation from the original Japanese manuscript into English.

### 2.2. Classification by Narcissistic Types

This study was based on the premise that behavioral strategies for maintaining positive self-evaluation differ depending on the factors of narcissism. Accordingly, participants were classified using the three subscales of the NPI-S (Narcissistic Personality Inventory Short version): Self-Assertion, Need for Attention and Praise, and Sense of Superiority and Competence. Because these subscales reflect different self-regulatory strategies that individuals use to maintain a positive self-evaluation, narcissistic types were classified into three types in this study based on these scores.

For each participant, total scores on the three subscales were calculated, and the subscale with the highest score was identified as the dominant characteristic, determining the participant’s narcissistic type. This classification method follows prior studies (Semba, 2017, 2018, 2024, 2025), which have demonstrated that individuals exhibit different response patterns under ego-threatening situations according to their dominant narcissistic factor.

### 2.3. Survey Items

The survey used in this study consisted of a face sheet and scales or items assessing narcissism, power harassment, ego threat, and supervisor and organizational support (see Appendix B). Except for the face sheet, all instruments used were developed or modified in Japan to ensure that the psychological states of workers were accurately assessed within the Japanese linguistic and cultural context.

#### 2.3.1. Face Sheet

This section asks respondents to indicate their gender, age, and occupation.

#### 2.3.2. Narcissism Scale

The NPI-S (Oshio, 1998, 1999) was used in this study. This instrument consists of 30 items and includes three subscales: Self-Assertion, Need for Attention and Praise, and Sense of Superiority and Competence (Oshio, 2004). The NPI-S is an adaptation of the original NPI (Raskin & Hall, 1979), modified to better reflect the Japanese cultural context. Participants rated each item on a five-point Likert scale ranging from 1 (does not apply at all) to 5 (applies very well). The total score for each subscale was calculated, and participants were classified into the narcissistic type corresponding to the subscale on which they scored the highest.

#### 2.3.3. Power Harassment Scale

To the best of the author’s knowledge, although several scales for measuring power harassment exist, none adequately capture the six types defined by the MHLW (2020): Physical Attack, Psychological Attack, Social Isolation, Excessive Demands, Insufficient Demands, and Invasion of Privacy. Accordingly, this study reviewed a prior study (Japan Institute for Women’s Empowerment & Diversity Management, 2011) and a government document (MHLW, 2020) to generate six behaviorally specific items corresponding to these types in ways appropriate to Japan’s legal and cultural context (see Table 1). All items were rated on a 10-point Likert scale ranging from 1 (strongly disagree) to 10 (strongly agree), with a higher score indicated a stronger tendency to engage in power harassment behaviors.

#### 2.3.4. Ego Threat Scale

The State Self-Esteem Scale developed by Heatherton and Polivy (1991) was used to measure ego threat. Following Tabata and Ikegami (2011), eight items from the performance and social subscales were selected, while items irrelevant to the study’s purpose (e.g., “I am concerned about my weight”) were excluded. This scale measures an individual’s self-evaluation at the present moment (M. Abe & Konno, 2007) and has been used in prior studies as a measure of ego threat (e.g., Watanabe & Karasawa, 2012).

Consistent with these prior studies, participants were instructed to restrict their responses to their current emotional state with the following: “The following questions are designed to assess what you are thinking right now. Please respond based on how you feel at this moment, rather than how you usually feel.” Items were rated on a five-point Likert scale ranging from 1 (does not apply) to 5 (applies). Higher total scores indicate greater perceived ego threat.

#### 2.3.5. Supervisor Support Scale

Supervisor support was measured using the Social Support Scale developed by Komaki (1994). This scale consists of 14 items and includes two subscales: emotional support and instrumental support. The total score across all items was used for analysis. Participants rated the support they received from their immediate supervisors on a five-point Likert scale ranging from 1 (strongly disagree) to 5 (strongly agree). Higher total scores indicate greater perceived supervisor support.

#### 2.3.6. Organizational Support Scale

Perceived organizational support was assessed using a Japanese translation of the Organizational Support Scale developed by Eisenberger et al. (1986). This scale measures employees’ beliefs regarding the extent to which their organization values their contributions and cares about their well-being. The scale has a one-factor structure, with several items sharing similar content. Therefore, considering the purpose of the present study and the response burden on participants, three items with high factor loadings were selected: “The company (organization) takes pride in my accomplishments at work” (loading: 0.76), “The company (organization) really cares about my well-being” (0.83), and “The company (organization) values my contributions to its well-being” (0.71). Participants rated on a seven-point Likert scale ranging from 1 (strongly disagree) to 7 (strongly agree). Higher total scores indicate greater perceived organizational support.

### 2.4. Statistical Analysis

First, basic descriptive statistics were calculated for the whole sample. Next, factor analyses using principal axis factoring with Promax rotation were conducted for all scales, and items deemed inappropriate were removed. Correlation analyses were then conducted among the variables included in the hierarchical multiple regression analyses for the ego-threatened group. Finally, hierarchical multiple regression analyses were conducted separately for each narcissistic group to examine (a) how the effects of ego threat and supervisor/organizational support on power harassment vary by narcissistic type, and (b) how the moderating effects of the two forms of support on the relationship between ego threat and power harassment vary by narcissistic type (see Figure 1).

Specifically, each of the power harassment items was entered as the dependent variable, and gender, age, occupation, ego threat, and the interaction terms of ego threat with supervisor and organizational support (ego threat × supervisor support, ego threat × organizational support, and ego threat × supervisor support × organizational support) were entered as independent variables.

To be more specific, in Step 1, gender, age, and occupation were entered as independent variables. In Step 2, ego threat, supervisor support, and organizational support were added. In Step 3, the interaction terms between ego threat and the support variables were included. To avoid multicollinearity, the interaction terms were centered following Aiken and West (1991). The significance level was set at 5%, and SPSS ver. 28 was used for the statistical analyses.

## 3. Results

### 3.1. Descriptive Statistics

Company A administered the questionnaire survey to a total of 1621 individuals and obtained 600 valid responses (valid response rate: 37.0%). The sample consisted of 300 men and 300 women. The mean age was 44.8 ± 13.4 years (men: 44.8 ± 13.5 years; women: 44.8 ± 13.4 years). Regarding occupational categories, 10 respondents (1.7%) were corporate or organizational executives/managers, 386 (64.3%) were company employees, 29 (4.8%) were public sector employees or government workers, 42 (7.0%) were self-employed or freelance workers, and 133 (22.2%) were part-time or temporary workers (see Table 2).

Next, factor analyses were conducted for all scales. Item Q5 of the State Self-Esteem Scale was excluded from further analyses due to its low communality value (0.046).

**Figure 2:**
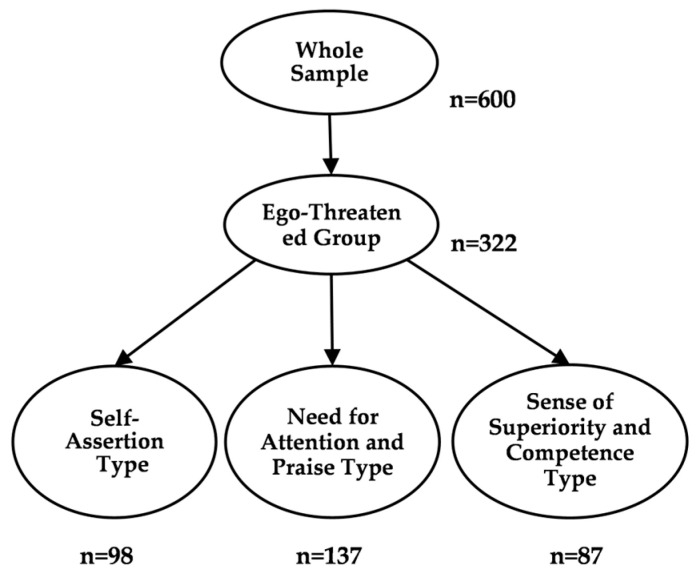
Grouping by narcissistic type.

Participants were then divided into high and low groups based on the median of the State Self-Esteem Scale (3.0). The high group, defined as the “ego-threatened group,” comprised 322 participants, including 98 of the Self-Assertion type, 137 of the Need for Attention and Praise type, and 87 of the Sense of Superiority and Competence type (see Figure 2).

### 3.2. Hierarchical Multiple Regression Analysis

To clarify how ego threat and support from supervisors and organizations influence power harassment across narcissistic types, and how such support moderates the relationship between ego threat and power harassment, hierarchical multiple regression analyses were conducted separately for each power harassment item by narcissistic type. Variance inflation factors (VIFs) were all below 3.4, indicating that multicollinearity was not a concern. The descriptive statistics and correlations of the variables included in the hierarchical multiple regression analysis are presented in Table 3.

The estimated results of the hierarchical multiple regression analyses for each item of power harassment by narcissistic type are presented in Table 4, Table 5 and Table 6. The following sections describe the results separately by narcissistic type.

#### 3.2.1. Self-Assertion Type

For the Self-Assertion type, the regression model predicting Social Isolation was significant at Step 2 (R^2^ = 0.169, *p* < 0.05), with perceived organizational support exerting a significant positive effect (β = 0.322, *p* < 0.05). Regarding Excessive Demands, the regression model was also significant at Step 2 (R^2^ = 0.170, *p* < 0.05), and organizational support showed a significant positive association (β = 0.373, *p* < 0.01). In addition, for Invasion of Privacy, the regression models were significant at both Step 2 and Step 3 (R^2^ = 0.215 and 240; *p* < 0.01 and *p* < 0.05, respectively), with organizational support demonstrating a significant positive effect at both steps (β = 0.359 and 0.399; *p* < 0.01).

#### 3.2.2. Need for Attention and Praise Type

For the Need for Attention and Praise type, the regression model predicting Physical Attack was significant at Step 1 (R^2^ = 0.098, *p* < 0.05), with gender showing a significant positive effect (β = 0.262, *p* < 0.01). For Psychological Attack, the regression models were significant across all steps (R^2^ = 0.096, 0.124, and 0.155; *p* < 0.05), and gender consistently exerted a significant positive effect (β = 0.251, 0.232, and 0.241; *p* < 0.01). Similarly, for Social Isolation, the model was significant at Step 1 (R^2^ = 0.094, *p* < 0.05), with gender positively predicting the outcome (β = 0.236, *p* < 0.01).

With respect to Excessive Demands, the regression models were significant across all steps (R^2^ = 0.191, 0.206, and 0.235; *p* < 0.001), and both gender (β = 0.237, 0.226, and 0.235; *p* < 0.01) and executive/managerial position (β = 0.344, 0.333, and 0.324; *p* < 0.001) showed significant positive effects. For Insufficient Demands, the models were significant at Step 1 and Step 2 (R^2^ = 0.103 and 0.122; *p* < 0.05), with gender exerting a significant positive effect (β = 0.260 and 0.245; *p* < 0.01). Finally, for Invasion of Privacy, the regression models were significant across all steps (R^2^ = 0.116, 0.133, and 0.160; *p* < 0.05), and gender consistently showed a significant positive association (β = 0.260, 0.248, and 0.250; *p* < 0.01).

#### 3.2.3. Sense of Superiority and Competence Type

For the Sense of Superiority and Competence type, the regression models predicting Physical Attack were significant at Step 2 and Step 3 (R^2^ = 0.191 and 0.395; *p* < 0.05 and *p* < 0.001, respectively). At Step 2, both gender (β = 0.259, *p* < 0.05) and organizational support (β = 0.354, *p* < 0.01) showed significant positive effects. At Step 3, being a public employee was negatively associated with Physical Attack (β = −0.232, *p* < 0.05), while ego threat × supervisor support had a significant positive effect (β = 0.415, *p* < 0.01).

For Psychological Attack, the regression models were significant across all steps (R^2^ = 0.157, 0.245, and 0.418; *p* < 0.05, *p* < 0.01, and *p* < 0.001, respectively). At Step 1, gender and executive/managerial position exerted significant positive effects (β = 0.223 and 0.244; *p* < 0.05). At Step 2, gender (β = 0.251, *p* < 0.05), executive/managerial position (β = 0.274, *p* < 0.05), and organizational support (β = 0.357, *p* < 0.01) were significant positive predictors. At Step 3, gender (β = 0.212, *p* < 0.05), executive/managerial position (β = 0.249, *p* < 0.01), and ego threat × supervisor support (β = 0.383, *p* < 0.01) showed significant positive effects, whereas public employee status (β = −0.213, *p* < 0.05) and supervisor support (β = −0.247, *p* < 0.05) exerted significant negative effects.

Regarding Social Isolation, the regression models were significant at Step 2 and Step 3 (R^2^ = 0.213 and 0.376; *p* < 0.05 and *p* < 0.001, respectively). At Step 2, gender and organizational support showed significant positive effects (β = 0.263 and 0.326; *p* < 0.05). At Step 3, supervisor support was negatively associated with Social Isolation (β = −0.239, *p* < 0.05), whereas ego threat × supervisor support had a significant positive effect (β = 0.339, *p* < 0.05).

For Excessive Demands, the regression model was significant at Step 3 (R^2^ = 0.313, *p* < 0.01), with ego threat × supervisor support exerting a significant positive effect (β = 0.294, *p* < 0.05). Similarly, for Insufficient Demands, the regression model was significant at Step 3 (R^2^ = 0.342, *p* < 0.001), and ego threat × supervisor support showed a significant positive association (β = 0.355, *p* < 0.05). Finally, for Invasion of Privacy, the regression models were significant at Step 2 and Step 3 (R^2^ = 0.221 and 0.374; *p* < 0.05 and *p* < 0.001, respectively). At Step 2, gender, executive/managerial position, and organizational support exerted significant positive effects (β = 0.252, 0.214, 0.343; *p <* 0.05, 0.05, 0.01), whereas at Step 3, ego threat × supervisor support showed a significant positive effect (β = 0.315, *p* < 0.05).

## 4. Discussion

The present study examined how ego threat and perceived support from supervisors and organizations influence workplace power harassment and how perceived support influences the relationships between ego threat and harassment across different narcissistic types, conceptualized as functionally distinct self-regulatory strategies. By moving beyond a unitary trait view of narcissism, this study provides a more nuanced understanding of when and for whom support serves as a buffer versus a trigger for aggressive workplace behavior. Overall, the findings demonstrate that the behavioral consequences of ego threat are not uniform but depend critically on how individuals regulate their self-worth and interpret social cues.

### 4.1. Theoretical Implications

The present results extend the framework of the threatened egotism model by showing that the role of social support is contingent on narcissistic strategy. For individuals classified as the Sense of Superiority and Competence type, supervisor support reduced certain forms of harassment under low-threatening conditions but paradoxically amplified aggressive behavior when ego threat was high. This pattern suggests that, for these individuals, support from a superior may be interpreted as a signal of diminished autonomy, competence, or status. Rather than functioning purely as a resource, support becomes a socially meaningful cue that highlights dependency and hierarchical asymmetry, thereby intensifying the need to restore threatened self-worth through dominance-oriented behavior.

In contrast, for the Self-Assertion type, organizational support was positively associated with behaviors such as Social Isolation and Invasion of Privacy. This finding implies that individuals who regulate self-worth through assertiveness and influence may interpret organizational endorsement as permission or legitimation to intervene in others’ domains. For the Need for Attention and Praise type, aggressive behaviors were more strongly associated with demographic and positional factors (e.g., gender and managerial status) than with support per se, suggesting that their behavior may be driven primarily by opportunities for visibility and control rather than by the symbolic meaning of support.

The findings of this study also suggest the need to critically reconsider the concept of “support” itself. Support in organizations is not merely a neutral resource, but a form of social interaction that carries different psychological meanings depending on how it is enacted and interpreted. For instance, superficial or formal support may be perceived as an implicit evaluation of employees’ competence or autonomy, potentially evoking ego threat. In contrast, support grounded in empathy, thoughtfulness, and authentic leadership may enhance employees’ sense of being valued within the organization and thereby mitigate ego threat. Therefore, to understand the conditions under which support functions as a protective factor and those under which it may transform into a psychological threat, further research focusing on the quality and modes of support is needed.

Together, these patterns refine ego threat theory by demonstrating that the same situational cue—support—can produce qualitatively different behavioral outcomes depending on the individual’s self-regulatory orientation. This person–situation interaction perspective aligns with recent calls in behavioral science to integrate personality processes with social–contextual meaning systems rather than treating traits and situations as independent predictors of behavior (Fleeson & Jayawickreme, 2015; Jayawickreme et al., 2019; Rauthmann et al., 2015).

The results of this study partially supported the proposed hypotheses. First, the results of this study did not support Hypothesis 1. Although a positive association between ego threat and power harassment was observed in some cases, no consistent significant relationship was found across all power harassment variables and narcissistic types. In contrast, support from supervisors and organizations was found to moderate the relationship between ego threat and power harassment for some combinations of power harassment variables and narcissistic types, partially supporting Hypotheses 2 and 3. Furthermore, the effects of ego threat and support on power harassment differed across narcissistic types, supporting Hypothesis 4. These findings suggest that support may function not only as a protective resource but also, under conditions of ego threat, as a situational cue that can intensify defensive dominance-oriented behavior.

### 4.2. Cultural and Contextual Significance

The Japanese organizational context provides a theoretically meaningful test case for examining these dynamics. In interdependent and hierarchical settings, evaluation by significant others carries heightened psychological weight. Support from superiors may therefore be experienced not only as assistance but also as implicit evaluation. The present findings suggest that such cultural configurations amplify the ambiguity of support and its potential to provoke compensatory dominance behavior under ego threat. Importantly, although the term power harassment is culturally specific, the underlying behavioral patterns—dominance, coercion, and intrusion under asymmetric power relations—are broadly relevant across organizational cultures. Thus, the results offer insights into how culturally embedded meaning systems shape the behavioral expression of threatened self-regulation.

### 4.3. Practical Implications

From a practical standpoint, the findings caution against assuming that increasing support will uniformly reduce workplace harassment. Support-based interventions may be effective for some employees but counterproductive for others, particularly those who rely on superiority-based narcissistic strategies under ego threat. Organizations should therefore move toward psychologically informed prevention strategies that consider individual differences in self-regulatory orientation.

For example, supervisory training programs should emphasize not only providing support but also how support is framed and communicated. For employees sensitive to autonomy and status, support that is autonomy-affirming rather than directive may be less likely to trigger defensive dominance behaviors. Additionally, harassment prevention efforts should incorporate assessment tools that identify narcissistic self-regulatory tendencies, allowing interventions to be tailored rather than uniformly applied.

### 4.4. Limitations and Future Directions

Several limitations should be noted. First, the cross-sectional design precludes strong causal inferences. Future research employing longitudinal or experimental designs could clarify the temporal dynamics between ego threat, support, and aggressive behavior. Second, all measures relied on self-report data. Although perceived support and perceived aggression are theoretically central constructs in ego threat processes, incorporating multi-source or behavioral measures would strengthen validity.

Third, the sample consisted only of Japanese employees, which enhances internal cultural coherence but limits generalizability. Cross-cultural replication would help determine the extent to which these dynamics operate similarly in less hierarchical or more individualistic contexts. Fourth, another limitation concerns the sampling method. As this study was conducted through a single online survey company, the representativeness of the sample may be limited. Participants were recruited from the company’s monitor panel, and thus characteristics of that panel—such as its recruitment procedures, demographic composition, and response tendencies—may have influenced the results. Therefore, caution is warranted when generalizing the findings of this study to the broader population of Japanese workers.

Finally, although narcissism was conceptualized as functionally distinct strategies, further work is needed to examine how these strategies develop over time and how they interact with organizational socialization processes.

## 5. Conclusions

The present study demonstrates that social support is not inherently protective but functions as an ambiguous social signal whose behavioral consequences depend on individual self-regulatory strategies and cultural context. By showing that support can facilitate power harassment under ego threat among individuals relying on superiority-based narcissistic strategies, this research refines ego threat theory and advances a functional understanding of narcissism in organizational settings. Importantly, this study addresses a critical gap in the literature by integratively examining how ego threat, perceived support, and narcissistic self-regulatory strategies interact to influence power harassment. While prior research has largely treated support as a uniformly beneficial resource, the present findings demonstrate that its effects are contingent and may even become counterproductive depending on individual and situational factors. By empirically identifying the dual role of support—as both a buffering resource and a potential trigger for defensive dominance—this study provides a more nuanced framework for understanding workplace aggression. Together, these findings underscore the importance of examining how well-intentioned social practices may produce unintended consequences in hierarchical and culturally embedded environments.

## Figures and Tables

**Table 1:** Six types of power harassment and related items.

Type of Power Harassment	Item
Physical Attack	I lost my temper and ended up using physical force. Examples: shaking, pushing, or hitting.
Psychological Attack	I sent an email (or similar message) that disparaged or denied a worker’s abilities, addressing not only the person but also other workers.
Social Isolation	I deliberately assigned a specific worker to a private room, preventing them from having contact with other workers.
Excessive Demands	I required a worker to perform menial tasks unrelated to their job, not allowing them to refuse, or forced them to participate in optional social gatherings or events.
Insufficient Demands	I assigned work that was significantly below the worker’s abilities or competence.
Invasion of Privacy	I inquired into a worker’s private relationships or friendships.

**Table 2:** Participant attributes.

		*n*	%
Sex	Male	300	50.0
	Female	300	50.0
	Total	600	100.0
Occupation	Executives/Manager	10	1.7
	Company Employee	386	64.3
	Public Employee/Government Workers	29	4.8
	Self-employed/Freelance Worker	42	7.0
	Part-time/Temporary Worker	133	22.2
	Total	600	100.0

**Table 3:** Descriptive statistics and correlations of variables used in the regression analysis (*n* = 322).

			M	SD	2	3	4
Power Harassment Variable	1a	Physical Attack	2.54	2.19	−0.065	−0.052 **	−0.200 **
	1b	Psychological Attack	2.41	2.15	−0.102	−0.084 **	−0.216 **
	1c	Social Isolation	2.36	2.16	−0.078	−0.082 **	−0.232 **
	1d	Excessive Demands	2.41	2.27	−0.075	−0.093 **	−0.235 **
	1e	Insufficient Demands	2.47	2.20	−0.076	−0.078 **	−0.213 **
	1f	Invasion of Privacy	2.43	2.12	−0.069	−0.117 ***	−0.261 **
Independent Variable	2	Ego Threat	3.58	0.54	-	−0.167 **	−0.215 **
	3	Supervisor Support	2.92	0.91		-	−0.587 **
	4	Organizational Support	3.30	1.36			-

** *p* < 0.01, *** *p* < 0.001.

**Table 4:** Main effects of ego threat and support on Physical Attack/Psychological Attack, and moderating effect of support (*n* = 322).

	Dependent Variable	Physical Attack									Psychological Attack								
		Self-Assertion Type (*n* = 98)			Need for Attention and Praise Type (*n* = 137)			Sense of Superiority and Competence Type (*n* = 87)			Self-Assertion Type (*n* = 98)			Need for Attention and Praise Type (*n* = 137)			Sense of Superiority and Competence Type (*n* = 87)		
Independent Variable		Step1	Step2	Step3	Step1	Step2	Step3	Step1	Step2	Step3	Step1	Step2	Step3	Step1	Step2	Step3	Step1	Step2	Step3
Sex (1-Female, 2-Male)		0.085	0.050	0.033	0.262 **	0.247 **	0.244 **	0.233 *	0.259 *	0.208	0.159	0.110	0.100	0.251 **	0.232 **	0.241 **	0.223 *	0.251 *	0.212 *
Age		0.098	0.105	0.100	−0.043	−0.034	−0.030	−0.017	−0.045	−0.082	−0.070	−0.069	−0.079	−0.067	−0.061	−0.049	0.038	−0.005	−0.038
Executives/Manager ^+^		−0.014	−0.011	−0.018	0.131	0.116	0.114	0.034	0.068	0.040	−0.016	−0.007	−0.010	0.134	0.115	0.106	0.244 *	0.274 *	0.249 **
Company Employee ^+^		0.248	0.197	0.194	0.069	0.079	0.063	0.021	−0.031	−0.014	0.185	0.130	0.135	0.068	0.076	0.051	0.087	0.034	0.046
Public Employee/Government Workers ^+^		0.034	0.029	0.038	0.070	0.073	0.069	−0.189	−0.228	−0.232 *	0.148	0.172	0.180	0.090	0.090	0.088	−0.155	−0.208	−0.213 *
Self-employed/Freelance Worker ^+^		0.055	−0.005	−0.015	−0.028	−0.028	−0.014	−0.114	−0.184	−0.108	−0.014	−0.043	−0.049	−0.015	−0.017	−0.012	−0.047	−0.112	−0.049
Ego Threat			−0.028	−0.071		−0.071	−0.102		0.060	−0.121		−0.102	−0.124		−0.110	−0.130		0.031	−0.147
Supervisor Support			−0.115	−0.137		−0.088	−0.133		−0.149	−0.203		0.034	0.012		−0.106	−0.164		−0.199	−0.247 *
Organizational Support			0.308 *	0.325 *		0.132	0.132		0.354 **	0.141		0.217	0.234		0.139	0.141		0.357 **	0.170
Ego Threat × Supervisor Support				0.150			0.114			0.415 **			0.104			0.241			0.383 **
Ego Threat × Organizational Support				−0.076			0.110			0.002			−0.098			−0.002			−0.025
Ego Threat × Supervisor Support × Organizational Support				0.101			0.144			0.225			0.037			0.156			0.235
*R* ^2^		0.065	0.128	0.137	0.098 *	0.117	0.137	0.100	0.191 *	0.395 ***	0.094	0.164	0.169	0.096 *	0.124 *	0.155 *	0.157 *	0.245 **	0.418 ***
Δ*R*^2^		0.065	0.063	0.009	0.098 *	0.019	0.020	0.100	0.092 *	0.203 ***	0.094	0.070	0.005	0.096 *	0.028	0.031	0.157 *	0.087 *	0.173 ***

* *p* < 0.05, ** *p* < 0.01, *** *p* < 0.001. ^+^ Part-time/Temporary Worker was set as the reference.

**Table 5:** Main effects of ego threat and support on Social Isolation/Excessive Demands, and moderating effect of support (*n* = 322).

	Dependent Variable	Social Isolation									Excessive Demands								
		Self-Assertion Type (*n* = 98)			Need for Attention and Praise Type (*n* = 137)			Sense of Superiority and Competence Type (*n* = 87)			Self-Assertion Type (*n* = 98)			Need for Attention and Praise Type (*n* = 137)			Sense of Superiority and Competence Type (*n* = 87)		
Independent Variable		Step1	Step2	Step3	Step1	Step2	Step3	Step1	Step2	Step3	Step1	Step2	Step3	Step1	Step2	Step3	Step1	Step2	Step3
Sex (1-Female, 2-Male)		0.101	0.048	0.020	0.236 **	0.218 *	0.226 *	0.247 *	0.263 *	0.192	0.054	−0.004	−0.032	0.237 **	0.226 **	0.235 **	0.272 *	0.291 *	0.215
Age		−0.080	−0.075	−0.088	−0.129	−0.125	−0.113	−0.027	−0.055	−0.086	−0.014	−0.004	−0.018	−0.087	−0.072	−0.059	−0.064	−0.093	−0.120
Executives/Manager ^+^		0.031	0.041	0.028	0.077	0.060	0.051	0.123	0.156	0.128	0.030	0.046	0.035	0.344 ***	0.333 ***	0.324 ***	0.105	0.125	0.097
Company Employee ^+^		0.165	0.100	0.104	0.068	0.075	0.050	0.095	0.046	0.075	0.176	0.106	0.107	0.038	0.052	0.027	0.044	0.008	0.037
Public Employee/Government Workers ^+^		0.177	0.191	0.213	0.093	0.092	0.091	−0.163	−0.211	−0.208	0.135	0.149	0.165	0.099	0.106	0.104	−0.162	−0.197	−0.193
Self-employed/Freelance Worker ^+^		0.005	−0.048	−0.061	−0.047	−0.050	−0.044	−0.049	−0.103	−0.014	0.031	−0.026	−0.042	−0.065	−0.061	−0.056	−0.041	−0.085	0.005
Ego Threat			−0.084	−0.134		−0.112	−0.129		0.076	−0.053		−0.080	−0.150		−0.023	−0.045		0.021	−0.097
Supervisor Support			−0.035	−0.077		−0.102	−0.162		−0.185	−0.239 *		−0.032	−0.075		−0.078	−0.133		−0.135	−0.186
Organizational Support			0.322 *	0.356 **		0.126	0.128		0.326 *	0.104		0.373 **	0.407 **		0.144	0.147		0.241	0.020
Ego Threat × Supervisor Support				0.246			0.243			0.339 *			0.258			0.230			0.294 *
Ego Threat × Organizational Support				−0.150			−0.007			0.108			−0.169			0.005			0.143
Ego Threat × Supervisor Support × Organizational Support				0.118			0.148			0.131			0.149			0.158			0.118
*R* ^2^		0.070	0.169 *	0.191	0.094 *	0.120	0.150	0.132	0.213 *	0.376 ***	0.039	0.170 *	0.195	0.191 ***	0.206 ***	0.235 ***	0.126	0.166	0.313 **
Δ*R*^2^		0.070	0.099 *	0.021	0.094 *	0.026	0.030	0.132	0.081	0.163 ***	0.039	0.131 **	0.025	0.191 ***	0.015	0.029	0.126	0.040	0.147 **

* *p* < 0.05, ** *p* < 0.01, *** *p* < 0.001. ^+^ Part-time/Temporary Worker was set as the reference.

**Table 6:** Main effects of ego threat and support on Insufficient Demands/Invasion of Privacy, and moderating effect of support (*n* = 322).

	Dependent Variable	Insufficient Demands									Invasion of Privacy								
		Self-Assertion Type (*n* = 98)			Need for Attention and Praise Type (*n* = 137)			Sense of Superiority and Competence Type (*n* = 87)			Self-Assertion Type (*n* = 98)			Need for Attention and Praise Type (*n* = 137)			Sense of Superiority and Competence Type (*n* = 87)		
Independent Variable		Step1	Step2	Step3	Step1	Step2	Step3	Step1	Step2	Step3	Step1	Step2	Step3	Step1	Step2	Step3	Step1	Step2	Step3
Sex (1-Female, 2-Male)		0.149	0.106	0.088	0.260 **	0.245 **	0.252 **	0.207	0.242 *	0.190	0.097	0.030	0.008	0.260 **	0.248 **	0.250 **	0.229 *	0.252 *	0.187
Age		0.049	0.057	0.041	−0.077	−0.070	−0.060	−0.036	−0.071	−0.103	−0.078	−0.069	−0.095	−0.066	−0.051	−0.043	−0.016	−0.045	−0.073
Executives/Manager ^+^		0.044	0.058	0.052	0.102	0.087	0.080	0.137	0.165	0.139	0.026	0.045	0.037	0.108	0.096	0.091	0.181	0.214 *	0.186
Company Employee ^+^		0.215	0.166	0.173	0.081	0.088	0.069	0.107	0.057	0.076	0.168	0.093	0.111	0.057	0.072	0.053	0.087	0.037	0.060
Public Employee/Government Workers ^+^		0.184	0.195	0.209	0.079	0.081	0.080	−0.110	−0.143	−0.144	0.162	0.186	0.209	0.085	0.091	0.088	−0.157	−0.200	−0.199
Self-employed/Freelance Worker ^+^		0.033	−0.004	−0.014	−0.062	−0.064	−0.058	−0.057	−0.132	−0.059	0.108	0.059	0.048	−0.133	−0.130	−0.118	−0.042	−0.105	−0.023
Ego Threat			−0.058	−0.098		−0.075	−0.110		0.012	−0.134		−0.109	−0.139		−0.045	−0.098		0.061	−0.077
Supervisor Support			−0.006	−0.042		−0.110	−0.143		−0.119	−0.168		0.018	−0.033		−0.050	−0.084		−0.165	−0.215
Organizational Support			0.260 *	0.288 *		0.134	0.139		0.339 **	0.142		0.359 **	0.399 **		0.134	0.140		0.343 **	0.131
Ego Threat × Supervisor Support				0.176			0.154			0.355 *			0.215			0.123			0.315 *
Ego Threat × Organizational Support				−0.158			0.049			0.034			−0.224			0.128			0.096
Ego Threat × Supervisor Support ×Organizational Support				0.070			0.164			0.173			0.038			0.206			0.157
*R* ^2^		0.086	0.155	0.169	0.103 *	0.122 *	0.142	0.108	0.187	0.342 ***	0.064	0.215 **	0.240 *	0.116 *	0.133 *	0.160 *	0.136	0.221 *	0.374 ***
Δ*R*^2^		0.086	0.069	0.014	0.103 *	0.020	0.019	0.108	0.080	0.155 **	0.064	0.151 **	0.025	0.116 *	0.017	0.027	0.136	0.086 *	0.152 **

* *p* < 0.05, ** *p* < 0.01, *** *p* < 0.001. ^+^ Part-time/Temporary Worker was set as the reference.

## Data Availability

The original data presented in the study are openly available in the Hiroshima University Institutional Repository at https://hiroshima.repo.nii.ac.jp/records/2041434 (accessed on 27 January 2026).

## Abbreviations

The following abbreviations are used in this manuscript:

MHLW	Ministry of Health, Labour and Welfare
NPI	Narcissistic Personality Inventory
NPI-S	Narcissistic Personality Inventory Short version

## Appendix A. Participant Information Statement

Request for Participation in a Questionnaire Survey

- Purpose of the Study and Request for Cooperation
  ➢ This study aims to examine the effects of support in organizations on power harassment in the workplace. The findings will be used for academic research contributing to the prevention of power harassment at work in the workplace.
  ➢ The survey consists of multiple-choice questions.
  ➢ Please read the following information carefully. We kindly ask for your cooperation in the survey only if you fully understand and agree to the terms described below.
- Voluntary Participation
  ➢ Participation in this study is entirely voluntary and based on your free will.
  ➢ The survey will take approximately 10 min to complete.
  ➢ You may choose not to participate or to discontinue participation at any time without any disadvantage.
  ➢ If you agree to participate, please select “I agree” in the initial consent question.
  ➢ You are not required to answer any questions you do not wish to respond to, and you may stop responding at any point.
- Protection of Personal Information
  ➢ All data will be managed using anonymous response IDs. Results will be published in academic conferences or journals; however, all data will be aggregated and analyzed statistically, and no personally identifiable information will be disclosed.
  ➢ The data will be strictly managed by the principal investigator and will not be used for any purpose other than this research.
- Contact Information

For inquiries regarding this study, please contact:

Dr. Ryoichi Semba

Principal Investigator

Department of Management, Kyoto Tachibana University

Email: semba@tachibana-u.ac.jp

Phone: 080-1990-6540

## Appendix B. Questionnaire

Note: Section 1 is the face sheet, Section 2 is the Narcissism Scale, Section 3 is the Ego Threat Scale, Section 4 is the Power Harassment Scale, Section 5 is the Supervisor Support Scale, and Section 6 is the Organizational Support Scale.

### Appendix B.1. Section 1

Thank you very much for your cooperation in this survey. We would like to ask you a few questions about yourself.

1. How old are you?       _____ years old
2. Please indicate your gender.   1 Female 2 Male
3. How many years have you been working at your current workplace? _____ years
4. Which of the following best describes your current position? Please select one option.

1 Staff	2 Senior Staff	3 Section Chief
4 Assistant Manager	5 Manager	6 General Manager
7 Executive Officer	8 Director	9 President

### Appendix B.2. Section 2

Please select one number that best applies to you and circle the number.

Example of a correct response

5 ④ 3 2 1

Example of an incorrect response

5 4○ 3 2 1

5 Applies very well
4 Somewhat applies
3 Neither applies nor does not apply
2 Somewhat does not apply
1 Does not apply at all

I think I am a person blessed with talent.	5 4 3 2 1
I think I have superior talent compared to others around me.	5 4 3 2 1
I think I am more capable than others.	5 4 3 2 1
I have the kind of talent that allows me to influence people around me.	5 4 3 2 1
I have strengths that others can learn from.	5 4 3 2 1
I think I am a person who can handle anything well.	5 4 3 2 1
People around me recognize my talent.	5 4 3 2 1
If I say something, people will believe it.	5 4 3 2 1
Because people around me say I am a good person, I think so myself.	5 4 3 2 1
It seems that everyone I interact with likes me.	5 4 3 2 1
I want to be popular with everyone.	5 4 3 2 1
I would rather be someone who attracts attention.	5 4 3 2 1
I want to be praised by everyone.	5 4 3 2 1
I want to be someone people talk about.	5 4 3 2 1
I have a desire to attract everyone’s attention.	5 4 3 2 1
I want to be respected by many people.	5 4 3 2 1
I feel uneasy if people around me do not think well of me.	5 4 3 2 1
I feel uneasy if people do not pay attention to me.	5 4 3 2 1
When the moment calls for it, I am willing to stand out and draw attention.	5 4 3 2 1
I want to become a person of great authority who can make others obey me.	5 4 3 2 1
I think I am a person with a strong sense of self-assertion.	5 4 3 2 1
I think I am someone who clearly expresses my opinions.	5 4 3 2 1
I think I am the complete opposite of a modest person.	5 4 3 2 1
I behave as I like without worrying about others, no matter the situation.	5 4 3 2 1
I like to make decisions on my own and take responsibility for them.	5 4 3 2 1
I often find myself becoming the center of attention while talking.	5 4 3 2 1
I think I am someone who takes on challenges in any situation.	5 4 3 2 1
I have lived the way I wanted to, and I want to continue doing so.	5 4 3 2 1
I think I am a person with a strong individuality.	5 4 3 2 1
I tend to stick to my own unique way of doing things.	5 4 3 2 1

### Appendix B.3. Section 3

This set of questions is designed to measure what you are feeling at this very moment. Please select one option that best reflects how you feel right now (not how you usually feel) and circle the corresponding number.

Example of a correct response

5 ④ 3 2 1

Example of an incorrect response

5 4○ 3 2 1

5 Applies
4 Somewhat applies
3 Neither applies nor does not apply
2 Somewhat does not apply
1 Does not apply

I feel as though I do not understand anything.	5 4 3 2 1
I am concerned about how I appear to others.	5 4 3 2 1
I feel disappointed in my own poor performance.	5 4 3 2 1
I feel that others see me as an unpleasant person.	5 4 3 2 1
I feel that I have considerable ability.	5 4 3 2 1
I am worried about how others perceive me.	5 4 3 2 1
I feel that nothing is going well for me right now.	5 4 3 2 1
At this moment, I feel that I am less attractive than others.	5 4 3 2 1

### Appendix B.4. Section 4

These questions ask about your behavior in the workplace over the past year. Do you think you have engaged in the following behaviors? Please circle the applicable number.

1 Strongly disagree
5.5 Neither agree nor disagree
10 Strongly agree

1. I lost my temper and ended up using physical force. Examples: shaking, pushing, or hitting.	1		2		3		4		5		6		7		8		9		10	
																			
2. I sent an email (or similar message) that disparaged or denied a worker’s abilities, addressing not only the person but also other workers.	1		2		3		4		5		6		7		8		9		10	
																			
3. I deliberately assigned a specific worker to a private room, preventing them from having contact with other workers.	1		2		3		4		5		6		7		8		9		10	
																			
4. I required a worker to perform menial tasks unrelated to their job, not allowing them to refuse, or forced them to participate in optional social gatherings or events.	1		2		3		4		5		6		7		8		9		10	
																			
5. I assigned work that was significantly below the worker’s abilities or competence.	1		2		3		4		5		6		7		8		9		10	
																			
6. I inquired into a worker’s private relationships or friendships.	1		2		3		4		5		6		7		8		9		10	
																			

### Appendix B.5. Section 5

We would like to ask you about your supervisors and senior colleagues in your workplace. Please circle the response that applies.

1. Strongly disagree
2. Somewhat disagree
3. Neither agree nor disagree
4. Somewhat agree
5. Strongly agree

1. Encourages me when I feel discouraged at work.	1 2 3 4 5
2. Is willing to talk with me about anything, from casual to serious matters.	1 2 3 4 5
3. Offers advice on what to do when I am having trouble with work-related problems.	1 2 3 4 5
4. Shows genuine concern and helps me think through what to do when I have personal worries or anxieties.	1 2 3 4 5
5. Regularly checks in on me.	1 2 3 4 5
6. Evaluates me fairly when I perform well at work.	1 2 3 4 5
7. Values me as a person and evaluates me highly.	1 2 3 4 5
8. Recognizes and acknowledges my abilities.	1 2 3 4 5
9. Provides knowledge and information that can be useful for my work.	1 2 3 4 5
10. Teaches me methods and tips for solving work-related problems.	1 2 3 4 5
11. Gives reliable advice regarding my work.	1 2 3 4 5
12. Willingly helps me when I have tasks that I cannot handle alone.	1 2 3 4 5
13. Takes care of tasks that must be done when I do not have enough time.	1 2 3 4 5
14. Helps me when my workload is extremely heavy.	1 2 3 4 5

### Appendix B.6. Section 6

Please answer the following questions about the organization you belong to.

1. Strongly disagree
2. Disagree
3. Slightly disagree
4. Neither agree nor disagree
5. Slightly agree
6. Agree
7. Strongly agree

1. The company (organization) takes pride in my accomplishments at work.	1 2 3 4 5 6 7
2. The company (organization) really cares about my well-being.	1 2 3 4 5 6 7
3. The company (organization) values my contributions to its well-being.	1 2 3 4 5 6 7
